# Mortality

**Published:** 1872-01-01

**Authors:** 


### MORTALITY.

MONTH OF DECEMBER, 1871.

| | |
|---|---|
| Accident—burned | 2 |
| " crushed | 1 |
| " asphyxia by coke gas | 2 |
| " boiler explosion | 4 |
| " fracture of skull | 2 |
| " " inferior maxilla | 1 |
| Accident—by fall | 3 |
| " run over by wagon | 1 |
| " gun-shot wound | 2 |
| " by railroad | 4 |
| " " suffocation | 1 |
| Abscess of thigh | 4 |
| Albuminuria | 1 |
| Amputation of leg | 1 |
| Apthæ | 1 |
| Apoplexy | 4 |
| Atheroma | 1 |
| Atelectasis pulman | 1 |
| Bladder, inflammation of | 1 |
| Bowels, obstruction of | 1 |
| " chronic disease of | 1 |
| Biliary calculi | 1 |
| Brain, congestion of | 7 |
| " inflammation of | 3 |
| " softening of | 1 |
| Bronchitis | 5 |
| " capillary | 4 |
| Cancer of breast | 2 |
| " ovarium | 1 |
| " of stomach | 1 |
| " of uterus | 1 |
| Cholera infantum | 1 |
| Consumption | 44 |
| Convulsion | 60 |
| " puerperal | 1 |
| Croup | 14 |
| " membranous | 3 |
| Debility | 3 |
| Delirium tremens | 2 |
| Diabetes | 2 |
| Diarrhœa | 2 |
| " chronic | 4 |
| Diphtheria | 16 |
| Dropsy, general | 4 |
| Dysentery | 3 |
| Embolia following phlebitis of leg | 1 |
| " of pelvic arteries | 1 |
| Enteritis | 5 |
| Entero colitis | 1 |
| " metritis | 1 |
| Exhaustion | 1 |
| Erysipelas | 4 |
| Exposure | 2 |
| Fever, congestive | 2 |
| " puerperal | 6 |
| " remittent | 6 |
| " scarlet | 24 |
| " " malignant | 1 |
| " typhoid | 57 |
| Gastritis | 4 |
| Hæmatemesis | 1 |
| Heart, disease of | 2 |
| " fatty degeneration of | 3 |
| " organic disease of | 2 |
| " rupture of | 1 |
| " valvular disease of | 2 |
| " neuralgia of | 1 |
| Hernia st an ulation | 2 |
| " | — |
| Hydrocephalus | 6 |
| Inanition | 5 |
| Intemperance | 1 |
| Intestines, disease of | 1 |
| Kidneys, tumor of | 1 |
| " Bright's disease of | 4 |
| Laryngitis | 2 |
| Lithiasis (grave) | 1 |
| Lungs, congestion of | 1 |
| Malformation (general) | 1 |
| " of head | 1 |
| Manslaughter | 1 |

| | |
|---|---|
| Measles | 1 |
| Meningitis | 9 |
| " cerebro-spinal | 3 |
| " tubercular | 2 |
| Metritis, puerperal | 1 |
| Metro peritonitis | 2 |
| Nephritis hepatitis | 1 |
| Old age | 9 |
| Paralysis | 1 |
| Peritonitis | 2 |
| " puerperal | 2 |
| Pneumonia | 31 |
| " typhoid | 3 |
| Potts' disease | 1 |
| Pyæmia | 1 |
| " and erysipelas | 1 |
| Rheumatism | 2 |
| Scrofula | 2 |
| Small-pox | 47 |
| Spine, injury of | 1 |
| Syphilis | 1 |
| Tabes mesenterica | 15 |
| Suicide by cutting throat | 1 |
| " " hanging | 1 |
| " " shooting | 1 |
| Throat, malignant sore | 1 |
| Uterus, hæmorrhage of | 2 |
| Uremic poisoning | 1 |
| Whooping cough | 4 |
| Total | 526 |
| Premature births | 6 |
| Still births | 47 |
| Total | 53 |

COMPARISON.

| | |
|---|---|
| Deaths in December, 1871 | 529 |
| " " preceding month in 1871 | 516 |
| Increase | 13 |

AGES.

| | |
|---|---|
| Under one year | 134 |
| One year to two | 32 |
| Two years to three | 36 |
| Three years to four | 11 |
| Four years to five | 18 |
| Five years to ten | 35 |
| Ten years to twenty | 29 |
| Twenty years to thirty | 62 |
| Thirty years to forty | 62 |
| Forty years to fifty | 48 |
| Fifty years to sixty | 17 |
| Sixty years to seventy | 27 |
| Seventy years to eighty | 13 |
| Eighty years to ninety | 5 |
| Total | 529 |
| Males | 284 |
| Females | 245 |
| Total | 529 |
| Married | 138 |
| Single | 241 |
| Total | 529 |
| Colored | 8 |
| White | 521 |
| Total | 529 |

NATIVITIES.

| | |
|---|---|
| Austria | 2 |
| Bohemia | 7 |
| Canada | 4 |
| Native Chicago | 61 |
| Foreign " | 172 |
| New York and other parts | 78 |
| Denmark | 5 |
| England | 18 |
| Germany | 89 |
| Holland | 2 |
| Ireland | 52 |
| Isle of Man | 1 |
| Norway | 12 |
| Poland | 1 |
| Scotland | 6 |
| Shetland Islands | 1 |
| Sweden | 11 |
| Switzerland | 2 |
| Unknown | 5 |
| Total | 529 |
| Deaths daily per month | 17 |
| Rain fell during month (inches) | 3.440 |

MORTALITY BY WARDS, DECEMBER, 1871.

| Wards. | No. Deaths. | Pop'n (1871). | | Percentage. |
|---|---|---|---|---|
| 1 | — | 8,103 | one death in | — |
| 2 | 2 | 13,449 | " " " | 13,449 |
| 3 | 33 | 17,934 | " " " | 543 |
| 4 | 11 | 14,022 | " " " | 1,275 |
| 5 | 15 | 14,991 | " " " | 999 |
| 6 | 37 | 22,918 | " " " | 619 |
| 7 | 48 | 15,590 | " " " | 325 |
| 8 | 54 | 25,420 | " " " | 471 |
| 9 | 49 | 30,778 | " " " | 628 |
| 10 | 14 | 17,292 | " " " | 1,235 |
| 11 | 21 | 19,212 | " " " | 772 |
| 12 | 17 | 15,018 | " " " | 883 |
| 13 | 14 | 9,740 | " " " | 696 |
| 14 | 12 | 9,339 | " " " | 779 |
| 15 | 77 | 25,706 | " " " | 334 |
| 16 | 15 | 16,380 | " " " | 1,092 |
| 17 | 13 | 18,814 | " " " | 1,449 |
| 18 | 12 | 18,805 | " " " | 1,567 |
| 19 | — | 9,237 | " " " | — |
| 20 | 1 | 14,522 | " " " | 14,522 |
| Accidents | 23 | — | | — |
| Barracks | 10 | — | | — |
| County Hosp. | 13 | — | | — |
| Foundling Home | 2 | — | | — |
| Home for Friendless | 2 | — | | — |
| Hosp. W'm'n & Chil. | 1 | — | | — |
| Manslaughter | 1 | — | | — |
| Police Station | 1 | — | | — |
| Mercy Hospital | 12 | — | | — |
| St. Jo. Orph. Asylum | 1 | — | | — |
| St. Luke's Hospital | 5 | — | | — |
| Scammon " | 2 | — | | — |
| Small-pox " | 8 | — | | — |
| Suicides | 3 | — | | — |
| Washington'n Home | 1 | — | | — |
| Total | 529 | | | |

